# The Hygiene of Infancy: Abstracts of Lectures

**Published:** 1869-01

**Authors:** George T. Elliot

**Affiliations:** Prof. of Obstetrics and the Diseases of Women and Children


### THE HYGIENE OF INFANCY:

### ABSTRACTS OF LECTURES DELIVERED AT THE BELLEVUE HOSPITAL MEDICAL COLLEGE,

By GEORGE T. ELLIOT, Jr., M.D.,
Prof. of Obstetrics and the Diseases of Women and Children.

Gentlemen:—The subject to which I shall call your attention, in the four lectures of this preliminary course, is of the first importance to the rational study and treatment of the diseases of infancy; for many of these owe their existence to infraction of the laws of hygiene.

To diminish the terrible—though to a certain extent inevitable—mortality of infancy, to avert evil influences, to develop the good, to diminish the necessity for drugs, and so to carry these helpless little ones through the perils of infancy that they may reach the less dangerous years of childhood with well-developed constitutions, are tasks which demand both the knowledge and the application of the best hygienic laws.

While it will be impossible for me to attempt to exhaust the subject, I shall avoid at least useless details, and endeavor to fix your minds only on what may be direct and practical; nor shall I hesitate to set the hygienic indications in a clearer light by illustrating pathological conditions which may follow their neglect, as well as those which may forbid success.

If time would permit our thorough study of the subject, we should commence with those hereditary predispositions and influences which affect for good or ill the fœtus and the man, and those conditions of the mother's health and hygiene which are liable to affect gestation; but, passing over these interesting questions, we come at once to a broad division of the subject.

I. DUTIES OF THE PHYSICIAN TO THE NEW-BORN CHILD.

*Establishment of Respiration.*—Hitherto, in the womb, it has drawn its supply of oxygen from the mother, through the placental circulation; now it is obliged to obtain this vitalizing agent from the outer world, through organs whose functions have rested in abeyance. Hence our first duty is to see that the function of respiration is fully established. Fully, I say, because it not unfrequently happens that unless this be thoroughly done, portions of the lungs are left unexpanded, collapsed as they were in the womb before respiration was necessary; and thus, sufficient machinery not being set in action, after a while oxygenation is not thoroughly accomplished; the respiration labors; the vital power fails; more lung tissue ceases to work, perhaps collapses; the surface becomes blue, the nerve tissue poisoned by black blood, the senses benumbed, the vital warmth displayed by the advancing coldness of death. This unexpanded condition of the air-cells, which may obtain from the failure to establish respiration, and to which the lungs of infants are liable to revert in conditions of debility and catarrh, is known under the name of atelectasis. Prevent these dangers by insuring such full and continued respirations as may make you morally certain that all the cells have been distended. Hearty and continued cries from the child generally attest this result.

Now children are often born in natural labor, and in labors attended by special dangers, in a condition of apparent death. A broad distinction is drawn by authors between those apparently dead or apparently dying, with a congested or a pallid surface of the body. Treatment has been formulated in accordance with these obvious signs. I do not dwell upon them. No greater congestion of the internal organs has ever come under my observation, in the autopsy of these children, than in cases where the surface has been pallid. Congestion of the skin does not kill; it is congestion and extravasation within that we dread. Skin congestion accompanies internal congestions, but these latter may exist without the former. Signs of strength and vigor may permit treatment contraindicated in premature and puny children. Do not believe that the liver and brain must be pallid, because the skin is white.

Do not assume that, because a child is born and shortly dies with a thoroughly congested and blue surface, it died from "the blue disease," or cyanosis. Cyanosis to constitute a disease, must be recurrent; or if believed to have caused the death under the circumstances we are considering, something more than a patent foramen ovale must be shown by the autopsy. The foramen ovale would be patulous, as a matter of course. How could it have closed in so short a time, even if its persistent patency were assigned as the cause of cyanosis?

When, therefore, children are born and do not respire, is blood to be let? Is the indication to be based on the color of the skin? What method is to be preferred? Shall we allow blood to flow from the cord, or take it by leeches? I mention the latter advice only for condemnation. If you allow the blood to flow from the cord, hold it well, as you would a cut axillary artery, so that you can control it at once. A teaspoonful is a limit beyond which I would very rarely go. But I very seldom allow any blood to flow, and still more rarely until I have rapidly tried the measures to which I now invite your attention. Establish respiration thoroughly, and the sluggish circulation becomes active, the ruddy glow of health colors the skin.

Free the mouth and nose from mucous and vaginal discharges. Note that there be no malformation. It has been noticed that a simple band of skin over both nostrils, easily divided with a bistoury, has powerfully affected the respiration of a new-born child. Women relatively breathe more with the thorax, men with the abdomen; perhaps the new-born child, destined in lactation to rely so much on the nostrils, may physiologically need them more than we. Free the nostrils and the mouth thoroughly, both in order to admit air, and because in the first inspirations these materials may be drawn into the air-passages and occlude the bronchi. Such conditions may have obtained in utero from premature inspiratory efforts. Liquor amnii and meconium may be demonstrated in the air-passages by the microscope. The child yet contained within the unbroken amniotic pouch, compelled to respire prematurely by reflex irritations, or by that respiratory need awakened by interference with the placental circulation, may thus be drowned in the womb of its mother, and the cause of death demonstrated at the autopsy. Try to prevent this accident to the respiratory passages when the child is born, and in your hands.

When the child is separated from the placenta, if the stimulus of the respiratory need, and the transition to the cool air of the room are not sufficient, spank it over the buttocks with the tips of your fingers, and rapidly use Marshall Hall's or Sylvester's method for the resuscitation of those who have been drawn from the water. If not promptly successful, plunge the body of the child in warm water (which should be ready in advance), and then into cold water. You thus keep up the warmth, draw blood to the surface, and increase the shock of the cold application. Spur the diaphragm and intercostals by brisk sprinkling of water; a lump of ice or a column of water to the epigastrium; then back again to the warm water, so as to diminish internal congestion and the benumbing influence of continued cold. From the warm water place the child on a blanket, on the floor or bed, and thoroughly try Hall's or Sylvester's method. I prefer Hall's but use both, and have seen children saved exclusively by each. It is not necessary to draw the tongue forward. It is important to keep the chin in a line with the sternum, and to keep the trachea somewhat prominent. Remember to prevent the child from getting too cold. Hot and cold water again. Slap, sprinkle, blow on the surface of the body, aid the slow and struggling expiration by gentle pressure on the chest. Have a battery on hand. Place the poles on the sides of the neck (third and fourth cervical), and over the diaphragm. The theory is to stimulate the phrenic nerve. The battery, however, under my observation, has proved less valuable than the other methods detailed, and I therefore only indicate the most important application. During this time abstraction of blood will have been considered. Do not let the water be too hot; you may scald the insensible child. Too hot water has been asserted to have caused trismus. Shall you inflate the lungs with your own breath? If so, be sure that the air enters the larynx. With skilful manipulation a catheter makes this certain. Generally the stomach is blown up, unless precautions be taken. Do not blow into the lungs so as to produce emphysema. I have seen emphysema, however, in new-born children, whose lungs had not been thus inflated. If you inflate the lungs, do not blow when the child is making a respiratory effort. In one word, my personal experience makes me rank this method of inflation as secondary to alternations of heat and cold, stimuli, and the methods of Hall and Sylvester.

Persist in these trials as long as the heart can be felt or heard, and a little longer than it can be heard. The quickest way to feel the heart is to put the pulp of your finger under the ribs and lift up the diaphragm. Pulsation can be felt thus when it cannot be touched through the thorax. Persist a while after the heart has apparently ceased to beat. A life for which you are responsible hangs upon the effort. There is nothing more surprising than the tenacity with which some infants cling to life, except the facility with which others lose it.

But, gentlemen, all your endeavors will often fail. For your satisfaction, and for the satisfaction of the family, obtain an autopsy. The pathology of fœtal life and of the still-born yields to none other in interest or value. It is a microcosm but too little explored. It is melancholy to see the neglect of the subject in practice and in the records of great hospitals. A still-born child one would suppose to be a child still-born from some unexplained and sufficient general cause. Start clear from such apathy, such delusions. The autopsy may show that you struggled against hope, that the establishment of respiration was hopeless, or its continuance impossible. Gather this consolation when you can. Search at least for truth. The respiratory passages may be proved by the autopsy to be absent in whole or in part. Trachea or bronchi may be replaced by impervious cords. Cysts, peritoneal effusions, pleuritic effusions, may have developed themselves in fœtal life, may not have killed the child, but may prevent air from reaching the lungs, the diaphragm from descending, the lungs from expanding. The pulmonary artery may be absent or barely pervious. The heart may be in front of the neck, within the abdomen, outside of the thorax; it may be unfitted for the strain of the altered circulation from malformation and from intra-uterine disease. The diaphragm may be open, and the intestines have crowded into the thorax and stopped the lungs from expanding. Extravasations on the brain and into its tissue may have caused the death. These extravasations may have occurred before the labor commenced. I have said enough to show that you may have the consolation of knowing and proving that your responsibility has been discharged, that the cause of death bore no relation to your management of the labor, or to your choice and use of means to establish respiration, when respiration was impossible.

*Ligation of the Cord.*—In ligating the cord, always examine the umbilicus thoroughly for hernial protrusion. Cut far enough away from the body to leave space for a second ligature, in case it becomes necessary to apply it after the occurrence of hemorrhage. The gelatinous material composing the envelope of the cord is very apt to make the first ligature slip.

Knots in the cord may be found, but they rarely produce death. Their occurrence has been explained by supposing that the head of the child passed down through a loop in the cord. The cord is often twisted about the neck; and it is sometimes necessary to use forceps to effect delivery in these cases; I have never, however, had to cut the cord before delivery. A cord shortened from this or other reasons may produce delayed labor; and, if the forceps be used, the resistance due to the cord may be felt upon attempting traction in increasing ratio to the advance. It is difficult or impossible to diagnosticate these cases, until the head is well down in the vagina, or until the head is delivered.

*Warmth and Ventilation.*—After having secured the establishment of respiration, it is of the first importance to see that the infant be kept warm. Of all the young mammals, the human probably requires the most care in this respect. Yet exceptional instances may be cited indicative of the opposite condition. Children exposed in the streets and taken to foundling hospitals often die from cold. The competent motherly nurse takes the greatest care of the warmth of the child. Sleeping with its mother is the natural means for warming the child, a species of incubation, but is attended with liability to accidents; the child may be smothered beneath the bedclothes, by the mother or nurse, either accidentally or intentionally, overlaying it. The mother or nurse is also very apt to nurse the child too often at night, and thus institute a bad habit both for herself and for the infant. Moreover, the air of the mother's bed is more or less impure from the lochia.

I now wish to advise you particularly to see that there is constantly in the nursery a sufficient supply of fresh air. No observations illustrate my remark better than those made in the Dublin Lying-in Asylum, where for twenty-five years the mortality was 1 in 6. On the introduction of proper ventilation, the mortality fell to 1 in 19⅓, and subsequently to 1 in 58½. A thousand cubic feet of space are ordinarily regarded as desirable for an adult; a young child requires no less than an adult. Apart from the respiratory troubles overcrowding produces, it increases the liability to epidemics, to ophthalmia, and depraved nutrition.

*Residence.*—Very frequently it will be found that a change of residence will prove of decided benefit to the infant, especially when some depressing or contagious atmospheric influence exists in the neighborhood where the child is residing. A change from one part of the city to another may be sufficient. Often, however, the sea-side or the mountain may offer special claims, especially for escape from heat.

*Urination.*—It is of great consequence to see that the infant passes its water. Urine is secreted and passed in utero, and may be passed during and just after birth. In some children the urine has been retained, and the distention of the bladder has been so great as to prove a cause of delayed labor. Cystic kidneys have done the same. In one case the bladder was found capable of containing two quarts of urine; in other cases it has ruptured before birth. After birth, almost before the child draws its first breath, it often passes its urine. Should it not do so within the first twenty-four hours, we should learn why not. It may happen that the bladder was emptied immediately after or during labor. It may be that so little milk has been taken that the kidneys have not been called upon to act freely. Babies urinate in direct proportion to the amount of milk or liquid nourishment they receive, in a ratio five or six times as great in proportion to bulk as in the case of the adult. Hence, whenever we learn that the infant is passing but a scanty amount of urine daily, it is always safe to ask whether it is receiving milk enough from its mother or the wet-nurse.

Obstruction to the passage of urine may occur from deformity, or the partial or total absence of the organs necessary to the function of urination; such as partial or complete absence of the urethra, absence of the bladder, with compensatory openings, or of the kidneys, or impervious ureters. Perhaps the bladder may be very capacious or atonic. A cause of obstruction to the flow of urine shortly after birth, in boys, is dependent upon simple agglutination of the urethral walls. (I have more frequently found urine in the bladders of still-born boys than in those of still-born girls. It is natural that it should be so.) This condition is easily remedied by the introduction of a silver probe, curved into the form of a catheter; the urine generally trickles out along its sides, and then flows freely. The reflex irritation thus produced is often all that is necessary. Whenever you are told that the water does not pass by the natural outlet, always examine thoroughly for some abnormal opening through which it may be passing unperceived, especially for vesico-vaginal fistula, cloacæ, and hermaphroditism. Sometimes, but not very frequently, a condition occurs, known as hydronephrosis, in which the bladder and ureters may be immensely dilated, so as to resemble the fœtal intestines, and the kidneys affected by the pressure of the retained urine. In one case under my observation, in which this condition was found, my explanation was that, owing to the shallowness of the pelvis and the obliquity of its brim, the bladder had fallen forwards, after dilation had commenced, thus producing an angular flexure of the urethra or neck of the bladder, preventing the discharge of the urine, for the urethra was normal in size; accumulation had then occurred, and, by the "back-water" action, produced the changes in the urinary tract, distending the ureters, calices, pelves, and causing absorption of the cortical structure. Retention of urine may also occur from pressure upon the ureter, as by the passage across it of a supernumerary branch of the renal artery. I have never seen a case in which puncture of the bladder was demanded, in the new-born child, for retention; but, if necessary, I should prefer the supra-pubic method.

*Cleanliness.*—The education of the infant should begin with the first days of its extra-uterine life, and a point of no little importance is to see that it does not lie in wet or soiled diapers. Let these be removed immediately after it has soiled them, and soon it will learn to indicate by its cries its disapproval of damp diapers. See that the napkins are not dried in crowded rooms before the registers. Moreover, if a child is allowed to lie almost constantly in its own excretions collected in the napkins, erythematous eruptions, or even ulcerations, will be formed upon its nates, and these may sometimes have a very suspicious appearance. Now, gentlemen, do not be in a hurry to diagnosticate all ulcerations you find upon the buttocks of an infant as necessarily syphilitic in character. Appearances should not always be interpreted against the infant. Uncleanliness, and neglect to apply other clean, dry napkins as soon as the first are soiled, is a very common source of sores about the infant's buttocks, simulating syphilitic cachectic ulcers. By removing the cause of the trouble, applying a mild lead wash or other lotion, and seeing that the child is well nourished, we can generally heal up these ulcerations without difficulty, and dissipate the mistaken diagnosis. In diarrhœa, redouble precautions: cleanliness, lead water, calamine powder, disinfectants.

*Passage of Fœces.*—The liquor amnii does not, as a rule, contain meconium. When the finger, introduced into the vagina, encounters this, its presence is commonly supposed to indicate the death of the fœtus, or a breech presentation. But even when there is no breech presentation, we should not lay too much stress upon this symptom when making our prognosis, except in so far as it is indicative of great danger to the fœtus. There are very few positive signs of death of the fœtus. Inability to recognize the fœtal heart-beat is not sufficient evidence that the child is dead. There are few very strong evidences of its death. No pulsation distinguishable, after a long lapse of time, in the cord; second, the perception by the finger that the parietal and occipital bones collapse and move about on pressure, while the skin peels off on friction. If you do not recognize by the touch that the child is putrid, try to deliver promptly, and revive it if possible.

Always inquire, the first day after birth, if the infant has had a passage from its bowels. If it has not, examine it carefully. An examination of the external orifice, alone, is not sufficient. Introduce a probe into the rectum, and see whether it does not end in a *cul-de-sac.* It may be that parts of the intestines, which you cannot reach, consist only of fibrous bands, and in these various contingencies the question will arise as to the formation of an artificial anus.

Obstruction of the intestinal canal may occur from infarction by an accumulation of epithelial scales.

In children born without an anus, there may be a connection of the rectum with the vagina or the bladder. In the former case, we should make an incision in the median line, establish an anus in its usual situation, and, later in life, heal the recto-vaginal fistula by the usual procedures. In the latter wait developments, or, if possible, follow the same course.

Simple closure of the raphe or lower part of the rectum is the easiest malformation to detect and treat. When the question arises as to the advisability of groping one's way with bistoury, scissors, and fingers, where the rectum ought to have been, and then of plunging a trocar into something above that we believe to be intestine, or when we select the alternative of an artificial anus, our duty is clear, to represent fully the uncertainties and dangers to the family, with the limited chance of success in the last contingency. If the parents refuse, a painful and unsatisfactory operation need not be performed. If they assent, or saddle you with the whole responsibility of the decision, you must even make the artificial anus, for it has saved life in the history of the operation, though you will probably fail. The alacrity to be felt in the operation is in direct ratio to the expectation of speedily reaching the intestine from below. Before performing it, wait for the intestine to be distended, if you cannot feel it, but not too long.—*N. Y. Medical Record.*

